# A Novel Approach to Word Sense Disambiguation Based on Topical and Semantic Association

**DOI:** 10.1155/2013/586327

**Published:** 2013-10-31

**Authors:** Xin Wang, Wanli Zuo, Ying Wang

**Affiliations:** ^1^College of Computer Science and Technology, Jilin University, Changchun 130012, China; ^2^School of Computer Technology and Engineering, Changchun Institute of Technology, Changchun 130012, China; ^3^Key Laboratory of Symbolic Computation and Knowledge Engineering of Ministry of Education, Changchun 130012, China

## Abstract

Word sense disambiguation (WSD) is a fundamental problem in nature language processing, the objective of which is to identify the most proper sense for an ambiguous word in a given context. Although WSD has been researched over the years, the performance of existing algorithms in terms of accuracy and recall is still unsatisfactory. In this paper, we propose a novel approach to word sense disambiguation based on topical and semantic association. For a given document, supposing that its topic category is accurately discriminated, the correct sense of the ambiguous term is identified through the corresponding topic and semantic contexts. We firstly extract topic discriminative terms from document and construct topical graph based on topic span intervals to implement topic identification. We then exploit syntactic features, topic span features, and semantic features to disambiguate nouns and verbs in the context of ambiguous word. Finally, we conduct experiments on the standard data set SemCor to evaluate the performance of the proposed method, and the results indicate that our approach achieves relatively better performance than existing approaches.

## 1. Introduction

Up to present, diverse WSD methods have been proposed. These methods are overviewed as machine learning (includes supervised and unsupervised) and external knowledge sources. Generally speaking, these methods have the potential bottleneck and limitation. However, almost all the methods, without exception, depend on the context in which the ambiguous word occurs. Moreover, the context size of the target word is too small to convey enough meaning for being disambiguated at a fine-grained level. More contexts which may not be necessarily helpful, on the contrary, will increase computational complexity. Consequently, in order to achieve disambiguation task, there are several challenges, as follows: (1) how to choose context, represent context, and determine context size for implementing all words disambiguation; (2) how to discover topic discriminative features from document for topic identification and implement disambiguation based on topical and semantic association. 

In this paper, we propose a novel approach aimed at disambiguating all words based on topical and semantic association. Our main contributions are the following: (1) combining topic chain and disambiguation context into topic semantic profile for identifying topic discriminative term and constructing topical graph based on the topic span intervals of topic discriminative term to implement the document's topic identification, (2) determining the unique sense of ambiguous term using topical-semantic association graph, paying more attention to exploiting syntactic features, semantic features, and topical features to implement verb and noun disambiguation. Finally, the evaluated experiments have been performed on the standard data set, and the results indicate our approach can achieve disambiguation task effectively.

## 2. Related Work

Word sense disambiguation is the ability to identify the words' sense in a computational manner [1]. We can broadly overview two main approaches to WSD, namely, machine learning and external knowledge sources. The former further distinguishes between supervised learning [2, 3] and unsupervised learning approach [4, 5], whereas the latter further divides into knowledge-based [6, 7] and corpus-based approaches [8]. These approaches based on the external resource usually have lower performance than the machine learning ways, but they have the advantage of a higher precision rate and a wider coverage. These approaches are overly dependent on the knowledge completeness and richness. Recently, some comprehensive approaches are becoming more and more prevalent, such as the integration of knowledge-based and unsupervised approach [9] and the integration of knowledge-based and corpus-based approach [10, 11]. In addition, the approach of domain-oriented disambiguation [12] is similar to our idea. The hypothesis of this approach is that the knowledge of a topic or domain can help disambiguate words in a particular domain text [1]. This approach achieves good precision and possibly low recall, due to the fact that particular domain information can be used to disambiguate mainly domain words, for example, in the domains of computer science, biomedicine [13, 14], tourism, and so on. Given all that, the major difference between our disambiguation strategy and these existing approaches is that we focus on term-concept association and concept-topic association, moreover, in the way of determining the appropriate size of disambiguation context. In addition, the verbs sense disambiguation is an important portion of WSD; Dligach and Palmer [15] propose a notion of Dynamic Dependency Neighbors (DDN) which takes noun as an object from a dependency-parsed corpus. Abend et al. [16] introduce a novel supervised learning model for mapping verb instances to VN classes, using rich syntactic features and class membership constraints. The above two methods are based on supervised learning methods with rich features based on part-of-speech tags, word stems, surrounding and cooccurring words, and dependency relationships.

## 3. Word Sense Disambiguation Based on Topical and Semantic Association

In this section, we introduce the description of word sense disambiguation in detail, which includes three core components, namely firstly, mapping a WordNet Sense to an ODP's Category Label for generating the term's topic semantic profile; secondly, extracting topic discriminative term through position feature, statistical feature, semantic feature, and topic span distribution feature, and leveraging topic discriminative terms for topic identification; finally, determining the unique sense of ambiguous term using topical-semantic association graph.

### 3.1. Mapping a WordNet Sense to an ODP's Category Label

We aim to construct a mapping relation from a WordNet sense to an ODP's category label. Our proposed approach effectively fuses the semantic knowledge with hierarchical topic category to generate topic semantic knowledge profile for expediently handling a series of research hot issues, such as information extraction, topic identification, and word sense disambiguation. 

For conveniences in describing follow-up contents, we give some basic terminologies.

**Definition 1 (topic chain)**

A topic chain (TC) is a branch of topic hierarchy and represents a sequence of ordered topic category label terms in ODP. It represents a notation of *t*_*m*_ > ⋯>*t*_2_ > *t*_1_, where *t*_1_ is a top topic term and *t*_*m*_ is a terminal topic term.

**Definition 2 (disambiguation context)**

A disambiguation context (DC) is a set of glosses, synonyms semantics, and hypernyms semantics for a term which may exist several senses in WordNet. DC represents the horizontal synonyms relation and the vertical hypernyms relation from lower-level concept to upper-level concept. Simultaneously, the glosses can also be available to calculate the semantic similarity.

**Definition 3 (topic semantic profile)**

A topic semantic profile (TSP), which characterizes term's semantic and its hierarchical topic category, is a sequence of 3-tuple and represents a notation of 〈*w*, DC, TC〉, where DC denotes the term *w*'s disambiguation context; TC denotes the term *w*'s topic chain label name.

Due to a variety of senses or the vague sense for a given term, the determination of its topic category label is the most difficult problem. In order to solve this problem, there are two significant aspects to be handled, one is to determinate a particular topic branch of term which is associated with multiple topics; the other is to assign the term's proper topic level, just in case too fine-grained hierarchical category to match the concept of user interests or information needs. Restricting semantic disambiguation context is a standard technique to mitigate the problem of term's cross topic. In addition, semantic similarity and cooccurrence information are also the ideal techniques for determining topic branch and topic level by pruning hierarchical category tree. During the process of our algorithm, we aim to determinate the mapping between WordNet and ODP. Formally, given the sense of a term in WordNet, we acquire a mapping to an ODP's topic chain as follows:

 
*f* (sense_term_) : Disambiguation  Context_WordNet_ → Topic  Chain_ODP_.

### 3.2. Leveraging Topic Discriminative Term for Topic Identification

#### 3.2.1. Identifying Topic Discriminative Term

**Definition 4 (topic discriminative term)**

Topic discriminative term (TDT) can be applied to characterize and highlight a term or phrase's related subject matters in the document. The term or phrase of high discriminative should be strongly associated with the semantic context, owns the number of sense as little as possible, and explicitly specifies the topical category. In addition, on account that technical terminology is monosemous in most cases, it can provide import clues for grasping the meaning and topic category.

**Definition 5 (the reoccurrence topic span of TDT)**

When a sentence is a basic processing unit, the reoccurrence topic span (TS) of TDT is defined as text spans for expressing the particular topical meaning, which starts form the first occurrence of TDT and ends to the last occurrence TDT. In this span interval, TDT may not appear in each sentence. Hence, the formulation of reoccurrence topic span interval (TSI) for a certain TDT is defined as [*S*_first_, *S*_last_], where the sentence identifiers of first and last occurrence are denoted as *S*_first_ and *S*_last_, respectively.

Then, we exploit position feature, statistical feature, semantic feature, and topic span distribution feature to identify and extract topic discriminative term. Intuitively, the term or phrase occurs in more special positions which include title, the first paragraph, the last paragraph, the first sentence, the last sentence, and so on. The more senses of term or phrase denote the weaker capacity of topic discrimination. The greater topic span distribution denotes the stronger topic representation. Consequently, we define the formula (1) for calculating the weight of TDT as follows:

(1) TDT(w)=α·∑i=1Tωi·tfi(w)+β·1|Senses(w)| +γ·On(w)·1+(Slast−Sfirst)||S||,

where *tf*_*i*_ represents term frequency which is the number of word occurrences in the *i*th type special position; *w*_*i*_ stands for the weight of the *i*th type special position; |Senses(*w*)| is the number of term senses in the WordNet and denotes preference to terms which select only a few topic categories; On(*w*) is the number of occurrences in the document; *S*_first_ and *S*_last_, respectively, denote the identifiers' number of sentences in the document; *S* is the total number of sentences in the document. The parameters of *α*, *β*, and *γ* are user-specified, the values of which are dynamically adjusted according to experimental effect.

#### 3.2.2. The Calculating Measure of Semantic Similarity in Hierarchical Structure

The hierarchical structure is the common characteristics in knowledge representation, such as the hypernym/hyponym relations in WordNet or the topic coverage in ODP. We utilize the hierarchical structure features for measuring the semantic similarity, that is, node depth and node distance. Intuitively, the deeper the depth of subsume, the greater their similarity. The node pair with the shorter distance between has the greater similarity than that of the pair with the longer distance between them. Assume

(2) Sim(Ci,Cj)=((1−λ)·e−α·l1+λ·e−α·l2)·eβ·h−e−β·heβ·h+e−β·h,

where *l*_1_ and *l*_2_ are respectively the shortest distance length from the node to the subsume; *h* is the depth of the subsume; the parameters *α*, *β*, and *λ* are in [0, 1].

For instance, given two topic chains TC_*i*_ and TC_*j*_ in ODP, the average similarity is measured their relatedness by using formula (3) as follows:

(3) $$\begin{array}{c} \text{Sim}\left(\text{T}{\text{C}}_{i},{\text{TC}}_{j}\right)=\frac{1}{\left|\text{T}{\text{C}}_{i}\right|\left|\text{T}{\text{C}}_{j}\right|}\underset{t_{a}\in {\text{TC}}_{i}}{\sum }\;\underset{t_{b}\in \text{T}{\text{C}}_{j}}{\sum }\text{Sim}\left(t_{a},t_{b}\right), \end{array}$$

where, *t*_*a*_ and *t*_*b*_, respectively, denote one of all terms in topic chains TC_*i*_ and TC_*j*_. 

#### 3.2.3. The Topic Identification Algorithm

To implement topic identification for a given document, we assume the following. (1) The reoccurrences of topic discriminative terms in a given document indicate the presence of a certain topic. (2) A topic similarity set of topic discriminative terms which occur in the text fragment will share the identical topic and similar semantic context. Intuitively, the longer reoccurrences of the TDTs are preferred over shorter ones. The more the TDTs in the certain text fragment are, the more chance there is that they are related to a similar topic content.

Formally, a document *D* is represented as a sequence of *n* sentences *S*_*i*_  (1 ⩽ *i* ⩽ *n*) which are the basic structure units. The *K* candidate topic discriminative terms distribute in these sentences and generate the *K* re-occurrence topic span intervals. A topical graph *G* = (*V*, *E*) is on undirected graph and may be consisted of *m* topical subgraphs. The vertices are represented for corresponding topic span intervals (TSI) of TDT; meanwhile, these TDTs associate with the corresponding topic semantic profiles which include disambiguation contexts and topic chains. The edges are connected according to the overlap relationship of topic span intervals of TDTs and the similarity relationship of TDTs' topic semantic profiles. In the process of generating topical graph, the subgraph is firstly constructed through immediate overlap of topic span intervals. Then, the multiple subgraphs are connected to the whole topical graph through the immediate adjoining relationship of topic span intervals of TDT, and these intervals are not overlapped.

Next, we need to determine the unique sense of candidate TDT which includes more than one topic semantic profile. In the topical graph, we begin from the vertices whose TDTs are monosemous and the higher weight, iteratively calculate the similarity of corresponding topic chains between current vertex and its neighbor ones through formula (4), and choose the topic chain of maximal similarity value as its neighbor vertex's topic chain, thereby, determine the unique topic semantic profile of the candidate TDT. Consider

(4) $$\begin{array}{c} \text{Sim}\left(\text{TDT},\text{TD}{\text{T}}^{\prime }\right)=\underset{\text{T}{\text{C}}_{i}\in \text{T}{\text{C}}_{\text{TDT}},\text{T}{\text{C}}_{j}\in \text{T}{\text{C}}_{\text{TD}{\text{T}}^{\prime }}}{\max}\text{Sim}\left(\text{T}{\text{C}}_{i},\text{T}{\text{C}}_{j}\right), \end{array}$$

where TDT is the current selected vertex; TDT′ is one of immediate neighbor vertices in the topical graph. The Sim(TC_*i*_, TC_*j*_) is calculated by formula xx.

After all candidate TDTs are determined by the unique topic semantic profile, we continue to update the weight of edges *W*_*ij*_ through formula (5) in the topical graph and prune completely irrelevant edges. Consider

(5) $$\begin{array}{c} W_{ij}=\{\begin{array}{cc} 0, & \text{where}\;\;\text{Sim}\left(\text{TD}{\text{T}}_{i},\text{TD}{\text{T}}_{j}\right)<\lambda ,\\ \text{Sim}\left(\text{TD}{\text{T}}_{i},\text{TD}{\text{T}}_{j}\right), & \text{where}\;\;\text{Sim}\left(\text{TD}{\text{T}}_{i},\text{TD}{\text{T}}_{j}\right)\geq \lambda . \end{array} \end{array}$$

The manipulation of pruning irrelevant edges also indicates the fact that there is a conflict between topic span intervals of two TDTs. Suppose that the edge between vertex TS_*i*_ and vertex TS_*j*_ is pruned. In order to describe the process of adjusting conflict interval, the topic span intervals of the TDT_*i*_ and TDT_*j*_ are represented as [*S*(*i*)_begin_, *S*(*i*)_last_] and [*S*(*j*)_begin_, *S*(*j*)_last_], respectively. The overlap relationship of the conflict interval includes two cases, namely, complete inclusion and partial intersection. Consider the following:

1. TSI(TDT_*j*_) ∈ TSI(TDT_*i*_): compared with TDT_*i*_, if the similarity value between other TDTs and TDT_*j*_ is greater than threshold *λ*, then the topic span interval of TDT_*i*_ is splitted into [*S*(*i*)_begin_, *S*(*j*)_begin_] and [*S*(*i*)_last_, *S*(*j*)_last_]; Otherwise, the vertex of the TDT_*j*_ is deleted.
2. TSI(TDT_*j*_)∩TSI(TDT_*i*_) ≠ *∅*: compared with TDT_*i*_, if the similarity value between other TDTs and TDT_*j*_ is greater than threshold *λ*, then the topic span interval of TDT_*i*_ is updated for [*S*(*i*)_begin_, *S*(*j*)_begin_] or [*S*(*j*)_last_, *S*(*i*)_last_]; Otherwise, the topic span interval of TDT_*j*_ is updated for [*S*(*j*)_begin_, *S*(*i*)_begin_] or [*S*(*i*)_last_, *S*(*j*)_last_].

In addition, if there do not exist other TDTs in the conflict interval, the split intervals have a bias for the greater weight of TDT.

On the basis of pruned topical graph, the document's topical describing information is formed through detecting the high-density components and choosing top-level cooccurrence topic concepts of topic chains. Firstly, the vertices of the highest degree centrality are chosen as the initial set for implementing the topical clustering. Secondly, the other vertices are iteratively integrated into the different topical clusters according to the adjacency relationship and the previous calculation result of similarity for topic chains. The isolated individuals and too small topical clusters will be ignored. Finally, owing to the fact that the conventional document tends to contain a relatively small number of topics, we focus on those higher density components and choose top-level cooccurrence topic concepts as document's topic category describing information. At the same time, the TDT associated with the bottom-level topical concept has its corresponding topic span intervals. These topic span intervals will be used to determine the appropriate size of context for the ambiguous term.

To achieve the whole process of leveraging topic discriminative term for topic identification, we will design the Algorithm 1.

### 3.3. Determining the Unique Sense of Ambiguous Term Using Topical-Semantic Association Graph

The occurrences of the ambiguous term in the different contexts clearly convey different senses, respectively. Meanwhile, a certain sense of the ambiguous target is associated with a particular topic, so that multiple senses can be distinguished through topical information. Consequently, we propose a topical-semantic association model that exploits the local feature and global feature in the context of ambiguous term to determine its unique sense. The local feature perspective is described through the syntactic clues and the semantic information of neighbor concept in the sentence level. The global feature perspective is characterized by topical association knowledge, namely, the topical describing information.

#### 3.3.1. The Representation of Context for the Ambiguous Term

The representation of ambiguous term in the context space is an important decisive factor for choosing the appropriate sense. In our approach, a series of related features are considered to represent the context. These features include syntactic features, semantic features, and topical features. The syntactic features are based on the preprocessing steps of the input text, such as tokenization, part-of-speech tagging, chunking, and parsing. The semantic features are with the help of topic semantic knowledge resources which map from WordNet to ODP in the first section. The topical features are based on the topical describing information from the above-mentioned preprocessing steps of topic identification.

The representation of context problem can be formally stated as follows.

- *A* is a list of the ambiguous terms in a portion of the text.
- *L* is a list of related terms around the ambiguous term *t*, these terms are topic discriminative terms or a fixed word sense.
- *S* is a list of topic semantic profile associated to the terms. It represents the semantic information of calculating the similarity.
- *T* is the topical describing information in each topic interval text fragment. It denotes the topical background knowledge of implementing disambiguation.

The above notations only appear in the appropriate context size. So, given the topical context *T*, the task for determining the unique sense of an ambiguous term *a*, is calculated by the function

(6) $$\begin{array}{c} f_{T}:\left\{A,L\right\}\times S\to ℜ. \end{array}$$

For the syntactic feature, each sentence is analyzed for the parse tree. In the tree structure, we parse all the syntactic units. For the target verb, we firstly distinguish a sentence which is the sentence frame and identify corresponding object and subject, respectively. For the target noun, we focus on the modifier structure, the parallel structure, and subordinate clause for subject or object. In this way, the notation of {*A*, *L*} is the target of verbs and nouns disambiguation, and represents 〈verb, noun〉, 〈adjective, noun〉, and 〈noun, noun〉 patterns.

#### 3.3.2. Constructing Topical-Semantic Association Graph

**Figure 1:**
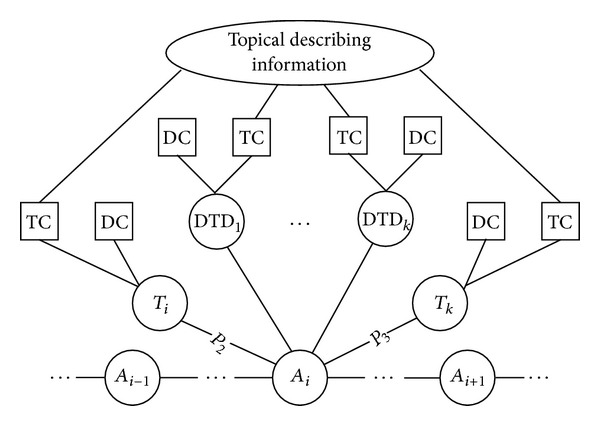
The topical-semantic association graph.

We fully exploit the interrelationships between topical graph and context space to construct topical-semantic association graph.  Figure 1 shows the example of the topical-semantic association graph. We take the proximal terms in the syntactic structure as adjoining feature, disambiguation context as semantic feature, and the topic chain of proximal terms and TDTs in topic span interval as topic feature. The constructing steps are as follows.

**Step 1**

On the basis of the syntactic preprocessing steps for the sentence *S*, all ambiguous terms {*A*_1_,…, *A*_*m*_} in the sentence *S* are linearly connected according to their occurrence in sequence.

**Step 2**

These ambiguous terms are taken as the centrality of topical-semantic association graph. Other terms {*T*_1_,…, *T*_*m*_} in the context space are connected to these targets according to the adjoining relationship.

**Step 3**

On syntactic parsing tree, the particular collocation patterns, namely, *P*_1_: 〈verb, noun〉, *P*_2_: 〈adjective, noun〉 and *P*_3_: 〈noun, noun〉, are annotated to the relations between terms.

**Step 4**

Suppose the sentence *S* belongs to *K* topic span intervals. TDTs {TDT_1_,…, TDT_*k*_} of these corresponding topic span intervals are connected to all ambiguous terms {*A*_1_,…, *A*_*m*_}.

**Step 5**

All terms' topic semantic profiles, namely disambiguation contexts and topic chains, are adhered to the corresponding terms. So, semantic contents of all terms in sentence *S* are integrated into topical-semantic association graph.

**Step 6**

The topic chain portions of all terms' topic semantic profiles are associated to the aforementioned topical describing information. So, the whole topical-semantic graph is constructed.

#### 3.3.3. Determining the Unique Sense through Choosing the Maximal Similarity

On the basis of the topical-semantic association graph, we focus on the disambiguation targets; firstly dispose the pattern of 〈noun, noun〉 and 〈adjective, noun〉 and then deal with the pattern of 〈verb, noun〉. The reason for this is that the task of disambiguating the nouns and noun phrases form are easy to implement through calculating the similarity of topic and semantic; nevertheless, the verb form is not suitable for directly calculating similarity. 

The basic idea of disambiguation for 〈noun, noun〉 is mainly a process of topic and semantic context comparison between a target term and other adjoining ones. In order to reduce the computation complexity, given a disambiguation target, we firstly judge whether the concepts of its topic chain appear in the topical describing information. If the topic concept occurs, then the branch of the corresponding topic chain is determined for the unique sense. Otherwise, the sense is fixed through choosing the semantic branch of the maximum similarity. The formula (7) can be defined as follows:

(7) f(Si)=arg max⁡si∈Sense(Ai)⁡(Sim(TCsi,TCT,TDT)+Sim(DCsi,DCT,TDT)),

where Sim(TC*s*_*i*_, TC_*T*,TDT_) and Sim(DC*s*_*i*_, DC_*T*,TDT_), respectively, denote the topic chain and disambiguation context similarity.

The treatment of verb sense disambiguation depends on three important clues, namely, the syntactic structure of sentence frame, the semantic information of synonyms, and the domain information of objects. The sentence frames state that different senses of verbs may occur with an infinitive, with a transitive, and with an intransitive syntactic frame. The syntactic structure information provided by WordNet is rather scarce and not enough to implement the task of verb disambiguation. Therefore, besides the above-mentioned sentence frame, WordNet also provides the form of 〈verb, domain〉 that characterizes the domain information of the verb associated object and a number of synonyms of a sense for target verb.

In front of disambiguating verbs sense, we extract major sectors about the verbs senses' semantic information from WordNet and harness the Lucene to index them according to the form of [verb  -  〈verb, domain〉  -  list_synonyms_  -  list_sentence  frame_  -  list_object_]. The first four contents are immediately obtained and the last part that records the list of objects is incrementally discovered through updating the indexing in the future.

The procedures of disambiguating the pattern of 〈verb, noun〉 are as follows. Firstly, compared with the syntactic structure of the target verb, the partial of senses may be filtered through sentence frames. Secondly, we calculate the similarity between the domain terms in the form of 〈verb, domain〉 and topic chains of the noun's object to choose the verb sense of maximum similarity. So, it is possible that the target verb has more than a sense. Finally, given a noun object, we can attain other synonyms verbs and retrieve the list of objects. If the result exists in the match content with object, then we choose the sense for the target verb.

## 4. Experimental Result

### 4.1. DataSet

In this section, we exploit the SemCor corpus to evaluate our approach. The SemCor corpus was the largest freely available textual corpus of semantically annotated words and has been extensively used in evaluating WSD systems.

### 4.2. Topic Identification Based on Extracting TDT

The key foundation of topic identification is the extraction of topic discriminative terms. We evaluate our topical identification algorithm using the precision (the number of correct documents over the number of all documents) on SemCor. We compare comprehensive features (All) with each feature, namely, statistical feature (TF, word frequency), positional and statistical feature (Pos + TF), semantic feature (SN, sense number), and topic spanning distribution feature (TS, topic spanning).

Table 1 summarizes the performance of extracting the topic discriminative terms based on the selection of different features. The columns “Mono” and “Poly,” respectively, show the results on the subset of monosemous and polysemous words, whereas column “All” shows results on all words. When the words are monosemous, semantic feature is the best results (91.0%); in contrast, positional + statistical feature and topic span distribution feature are better than semantic feature (80.8% and 83.1%). Let us continue to concentrate on the results we obtained with comprehensive features. As can be seen, all measures of comprehensive features perform better than the each feature. Especially, topic span distribution feature (86.2%) plays a more important role for improving the accuracy rate of documents' topic identification. Next, we further analyze the main failure reason of the topic identification. It is due to the fact that there are not higher degree centrality vertices in topic graph. This often degrades performance, as too many low-degree centrality vertices may lead to more difficulty in identify the document's topic. In addition, the probable cause is to determine the improper unique topic semantic profile of the candidate TDT.

### 4.3. The Performance of Word Sense Disambiguation

We compare our WSD approach based on topical and semantic association (TSA) using WordNet + ODP with other state-of-the-art WSD approaches, namely, the ExtLesk algorithm and the SSI algorithm. In addition, we evaluate separately the performance on nouns only, verbs only, and all words.

Table 2 indicates that the result of TSA with WordNet+ODP achieves the best performance to disambiguate words. The performances obtained for nouns are sensibly higher than the one obtained for verbs, confirming the claim that topical describing information is crucial to determine the unique sense of ambiguous term. On the nouns-only subsection of the result, the performance of TSA is comparable with SSI and significantly is better than other state-of-the-art algorithms (+2.6% F1 against SSI).

## 5. Conclusions

In this paper, we propose a novel approach for word sense disambiguation based on topical and semantic association. Our experiments show that the topic categories of Open Directory Project merged into WordNet are of high quality and, more importantly, it enables external knowledge-based WSD applications to perform better than the existing methods of only using WordNet. In addition, we also find that the applied topical and semantic association into determining the unique sense obviously influences WSD performance. We obtain a large improvement when adopting the WSD algorithm based on topical-semantic association graph.

## Figures and Tables

**Algorithm 1:**
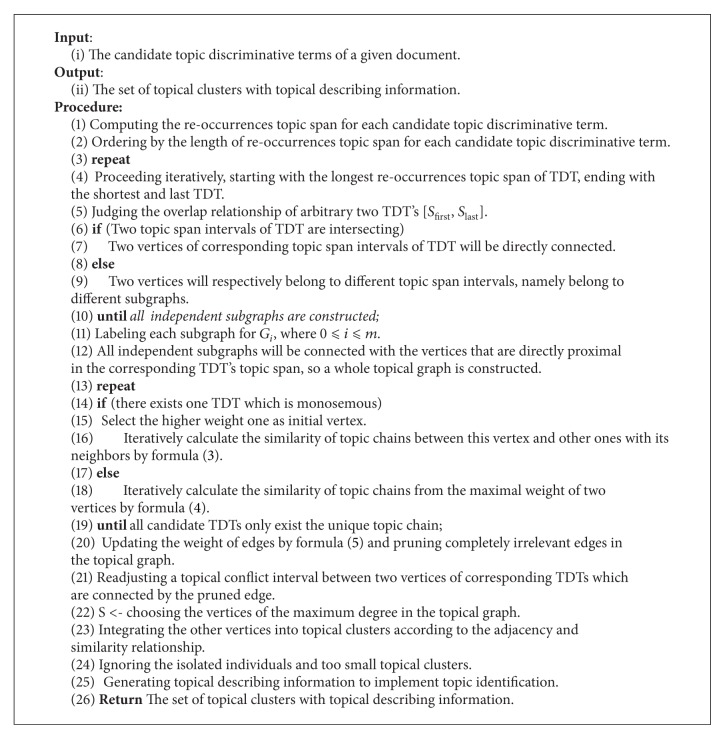
Leveraging Topic Discriminative Term for Topic Identification.

**Table 1:** The performance of the topic identification based on extracting topic discriminative terms.

Measure feature	Mono.	Poly.	All
TF	78.3	76.1	78.0
Pos + TF	82.1	80.8	81.6
SN	**91.0**	65.5	74.0
TS	88.6	83.1	86.2
All	**92.4**	**84.6**	**87.8**

**Table 2:** The performance of disambiguating through TSA versus other state-of-the art algorithms.

Algo.	Nouns only			Verbs only			All words		
	*P*	*R*	*F*1	*P*	*R*	*F*1	*P*	*R*	*F*1
TSA	82.5	68.9	75.1	69.3	57.7	63.0	76.8	60.2	67.4
ExtLesk	80.5	62.1	70.1	60.7	47.9	53.5	71.5	50.9	59.5
SSI	81.6	65.2	72.5	63.5	54.6	58.7	74.0	58.7	65.5
MFS	74.7	74.7	74.7	59.1	59.1	59.1	68.4	68.4	68.4
